# Supplementary material for: Changes in urgent and emergency care activity associated with COVID-19 lockdowns in a sub-region in the East of England: Interrupted times series analyses
Source: PLoS One. 2024 Nov 1;19(11):e0311901. doi: 10.1371/journal.pone.0311901 (PMC11530045; doi:10.1371/journal.pone.0311901)
Supplement: S1 Table — P<0.001 for all comparisons between the three periods with ANOVA test. ED emergency department. SD standard deviation. (DOCX) [file pone.0311901.s001.docx]

**S1 Table.** **Mean values of urgent and emergency care indicators in each hospital before, during and after COVID lockdown periods.** P<0.001 for all comparisons between the three periods with ANOVA test. ED emergency department. SD standard deviation

|  | Norfolk and Norwich University Hospital (NNUH) | | | James Paget \|University Hospital (JPUH) | | | Queen Elizabeth Hospital  (QEH) | | |
| --- | --- | --- | --- | --- | --- | --- | --- | --- | --- |
| Variable | Pre-COVID | Lockdown | Post-lockdown | Pre-COVID | Lockdown | Post-lockdown | Pre-COVID | Lockdown | Post-lockdown |
| Mean no (SD)^1^ per day | | | | | | | | | |
| All visits | 314.30 (40.14) | 288.20 (46.30) | 333.29 (32.43) | 192.33 (23.54) | 167.93 (29.89) | 201.23 (25.47) | 143.51 (16.45) | 126.12 (22.47) | 158.33 (18.76) |
| Arrive by ambulance | 127.39 (11.70) | 128.87 (14.52) | 105.36 (17.54) | 61.21 (8.47) | 58.46 (8.07) | 54.80 (9.23) | 46.11 (6.72) | 47.43 (7.40) | 45.00 (7.29) |
| Arrive by other means | 186.90 (35.38) | 159.33 (36.88) | 227.92 (31.98) | 131.11 (21.36) | 109.47 (25.86) | 146.43 (23.65) | 97.40 (14.30) | 78.69 (18.57) | 113.33 (17.79) |
| Injuries | 96.66 (16.20) | 82.84 (18.95) | 102.06 (18.03) | 55.01 (11.08) | 45.42 (13.88) | 56.22 (12.23) | 43.30 (8.58) | 37.64 (10.23) | 46.35 (9.40) |
| Circulatory disease | 25.91 (5.84) | 27.82 (6.84) | 28.25 (6.09) | 10.89 (3.55) | 9.25 (3.49) | 11.28 (3.62) | 12.66 (3.81) | 13.58 (4.52) | 17.35 (5.10) |
| Referred by primary care | 32.40 (17.22) | 35.03 (18.54) | 46.45 (21.63) | 7.45 (4.49) | 4.16 (2.61) | 5.58 (4.12) | 15.19 (9.27) | 11.56 (7.60) | 15.24 (9.77) |
| Referred by NHS 111 | 33.46 (11.57) | 39.52 (15.78) | 28.09 (7.63) | 5.17 (3.34) | 6.20 (4.24) | 7.88 (3.90) | 13.66 (7.24) | 12.58 (6.87) | 9.93 (4.78) |
| Mean (SD) | | | | | | | | | |
| Time at ED (minutes) | 252.81 (43.05) | 270.22 (44.63) | 364.96 (65.50) | 178.06 (30.85) | 164.10 (30.84) | 259.95 (65.02) | 204.69 (38.72) | 179.03 (32.74) | 300.13 (83.48) |
| Time in ED (Ambulance arrivals) | 332.00 (72.21) | 361.57 (71.07) | 586.66 (151.47) | 239.72 (55.97) | 219.65 (58.96) | 398.58 (144.14) | 294.26 (70.55) | 249.65 (57.37) | 504.18 (184.58) |
| Time in ED (Other arrivals) | 198.70 (31.98) | 193.41 (29.22) | 267.40 (50.26) | 148.99 (25.60) | 133.40 (24.21) | 209.44 (51.54) | 161.86 (28.65) | 135.21 (22.45) | 221.78 (58.63) |
| Ambulance handover > 60 mins (%) | 7.60 (9.24) | 2.29 (4.59) | 23.16 (16.86) | 2.76 (5.83) | 3.66 (6.81) | 23.99 (18.86) | 13.24 (13.09) | 6.63 (10.40) | 26.89 (18.56) |
