# Supplementary material for: Changes in urgent and emergency care activity associated with COVID-19 lockdowns in a sub-region in the East of England: Interrupted times series analyses
Source: PLoS One. 2024 Nov 1;19(11):e0311901. doi: 10.1371/journal.pone.0311901 (PMC11530045; doi:10.1371/journal.pone.0311901)
Supplement: S2 Table — Linear regression models, adjusted for day of week and month of year (model 1). NNUH Norfolk and Norwich University Hospital. JPUH James Paget University Hospital. QEH Queen Elizabeth Hospital. CI confidence interval. (DOCX) [file pone.0311901.s002.docx]

**S2 Table. Changes in mean number of daily emergency department attendances in each hospital from pre-COVID period to COVID lockdown period, and from pre-COVID period to post-lockdown period.** Linear regression models, adjusted for day of week and month of year (model 1). NNUH Norfolk and Norwich University Hospital. JPUH James Paget University Hospital. QEH Queen Elizabeth Hospital. CI confidence interval

| **Hospitals and variables** | **Coefficient** | | **95% CI** | **p-value** | **Coefficient** | **95% CI** | **p-value** | **Coefficient** | **95% CI** | **p-value** |
| --- | --- | --- | --- | --- | --- | --- | --- | --- | --- | --- |
|  | **All visits** | | | | **Ambulance arrival** | | | **Non-ambulance arrival** | | |
| ***NNUH*** |  | |  |  |  |  |  |  |  |  |
| Intercept (pre-COVID mean) | 279.72 | | (272.78, 286.65) | <0.001 | 126.18 | (123.23, 129.13) | <0.001 | 153.54 | (147.50, 159.58) | <0.001 |
| Change pre-COVID to lockdown | -25.96 | | (-30.24, -21.69) | <0.001 | 1.54 | (-0.28, 3.36) | 0.10 | -27.50 | (-31.23, -23.78) | <0.001 |
| Change pre-COVID to post-lockdown | 19.12 | | (15.61, 22.62) | <0.001 | -21.98 | (-23.47, -20.48) | <0.001 | 41.09 | (38.04, 44.14) | <0.001 |
| ***JPUH*** |  | |  |  |  |  |  |  |  |  |
| Intercept (pre-COVID mean) | 175.74 | | (170.96, 180.52) | <0.001 | 60.43 | (58.70, 62.17) | <0.001 | 115.30 | (111.09, 119.52) | <0.001 |
| Change pre-COVID to lockdown | -24.36 | | (-27.31, -21.41) | <0.001 | -2.75 | (-3.82, -1.67) | <0.001 | -21.62 | (-24.22, -19.02) | <0.001 |
| Change pre-COVID to post-lockdown | 8.95 | | (6.53, 11.36) | <0.001 | -6.40 | (-7.28, -5.52) | <0.001 | 15.35 | (13.22, 17.48) | <0.001 |
| ***QEH*** |  | |  |  |  |  |  |  |  |  |
| Intercept (pre-COVID mean) | 130.33 | | (126.91, 133.76) | <0.001 | 44.79 | (43.37, 46.21) | <0.001 | 85.55 | (82.61, 88.48) | <0.001 |
| Change pre-COVID to lockdown | -17.34 | | (-19.45, -15.22) | <0.001 | 1.33 | (0.46, 2.21) | 0.003 | -18.67 | (-20.48, -16.86) | <0.001 |
| Change pre-COVID to post-lockdown | 14.86 | | (13.13, 16.59) | <0.001 | -1.10 | (-1.82. -0.38) | 0.003 | 15.96 | (14.47, 17.44) | <0.001 |
|  | **Injury** | | | | **Circulatory disease** | | | **Referred by primary healthcare team** | | |
| ***NNUH*** |  | |  |  |  |  |  |  |  |  |
| Intercept (pre-COVID mean) | 84.31 | | (81.23, 87.38) | <0.001 | 25.23 | (24.04, 26.43) | <0.001 | 36.15 | (33.74, 38.55) | <0.001 |
| Change pre-COVID to lockdown | -13.80 | (-15.70, -11.91) | | <0.001 | 1.91 | (1.18, 2.65) | <0.001 | 2.67 | (1.19, 4.15) | <0.001 |
| Change pre-COVID to post-lockdown | 5.42 | (3.87, 6.97) | | <0.001 | 2.34 | (1.73, 2.94) | <0.001 | 14.00 | (12.79, 15.22) | <0.001 |
| ***JPUH*** |  |  | |  |  |  |  |  |  |  |
| Intercept (pre-COVID mean) | 50.92 | (48.73, 53.10) | | <0.001 | 11.12 | (10.40, 11.83) | <0.001 | 7.43 | (6.66, 8.20) | <0.001 |
| Change pre-COVID to lockdown | -9.57 | (-10.92, -8.22) | | <0.001 | -1.64 | (-2.09, -1.20) | <0.001 | -3.38 | (-3.86, -2.89) | <0.001 |
| Change pre-COVID to post-lockdown | 1.23 | (0.12, 2.33) | | 0.03 | 0.39 | (0.03, 0.75) | 0.04 | -1.69 | (-2.08, -1.30) | <0.001 |
| ***QEH*** |  |  | |  |  |  |  |  |  |  |
| Intercept (pre-COVID mean) | 38.07 | (36.42, 39.73) | | <0.001 | 11.69 | (10.82, 12.55) | <0.001 | 18.71 | (17.48, 19.94) | <0.001 |
| Change pre-COVID to lockdown | -5.63 | (-6.65, -4.61) | | <0.001 | 0.92 | (0.39, 1.45) | <0.001 | -4.03 | (-4.80, -3.26) | <0.001 |
| Change pre-COVID to post-lockdown | 3.07 | (2.23, 3.90) | | <0.001 | 4.69 | (4.26, 5.13) | <0.001 | -0.44 | (-1.07, 0.19) | 0.17 |
|  | **Referred by NHS111** | | | |  |  |  |  |  |  |
| ***NNUH*** |  |  | |  |  |  |  |  |  |  |
| Intercept (pre-COVID mean) | 25.10 | (23.24, 26.97) | | <0.001 |  |  |  |  |  |  |
| Change pre-COVID to lockdown | 6.14 | (4.99, 7.29) | | <0.001 |  |  |  |  |  |  |
| Change pre-COVID to post-lockdown | -5.29 | (-6.23, -4.34) | | <0.001 |  |  |  |  |  |  |
| ***JPUH*** |  |  | |  |  |  |  |  |  |  |
| Intercept (pre-COVID mean) | 4.43 | (3.69, 5.16) | | <0.001 |  |  |  |  |  |  |
| Change pre-COVID to lockdown | 1.02 | (0.57, 1.48) | | <0.001 |  |  |  |  |  |  |
| Change pre-COVID to post-lockdown | 2.73 | (2.36, 3.10) | | <0.001 |  |  |  |  |  |  |
| ***QEH*** |  |  | |  |  |  |  |  |  |  |
| Intercept (pre-COVID mean) | 11.27 | (10.28, 12.25) | | <0.001 |  |  |  |  |  |  |
| Change pre-COVID to lockdown | -1.10 | (-1.71, -0.49) | | <0.001 |  |  |  |  |  |  |
| Change pre-COVID to post-lockdown | -3.71 | (-4.21, -3.21) | | <0.001 |  |  |  |  |  |  |
