# Supplementary material for: Changes in urgent and emergency care activity associated with COVID-19 lockdowns in a sub-region in the East of England: Interrupted times series analyses
Source: PLoS One. 2024 Nov 1;19(11):e0311901. doi: 10.1371/journal.pone.0311901 (PMC11530045; doi:10.1371/journal.pone.0311901)
Supplement: S5 Table — Linear regression models adjusted for day of week and month of year (model 2). NNUH Norfolk and Norwich University Hospital. JPUH James Paget University Hospital. QEH Queen Elizabeth Hospital. CI confidence interval. (DOCX) [file pone.0311901.s005.docx]

**S5 Table. Change in level and change in slope (continuous increase or decrease per year) in minutes waiting at emergency departments in each hospital, during pre-COVID, lockdown and post-lock periods.** Linear regression models adjusted for day of week and month of year (model 2). NNUH Norfolk and Norwich University Hospital. JPUH James Paget University Hospital. QEH Queen Elizabeth Hospital. CI confidence interval

| **Outcome** | **All visits** | | | **Ambulance arrival** | | | **Non-ambulance arrivals** | | |
| --- | --- | --- | --- | --- | --- | --- | --- | --- | --- |
| **Hospitals and variables** | **Coefficient** | **95% CI** | **p-value** | **Coefficient** | **95% CI** | **p-value** | **Coefficient** | **95% CI** | **p-value** |
| ***NNUH*** |  |  |  |  |  |  |  |  |  |
| Slope pre-COVID | 41.49 | (35.06, 47.91) | <0.001 | 73.96 | (61.88, 86.05) | <0.001 | 31.17 | (26.13, 36.22) | <0.001 |
| Change in level pre-COVID to lockdown | -53.19 | (-99.32, -7.06) | 0.02 | -84.23 | (-171.00, 2.53) | 0.06 | -34.21 | (-70.42, 1.99) | 0.06 |
| Change in slope pre-COVID to lockdown | 3.46 | (-15.20, 22.12) | 0.72 | 1.38 | (-33.72, 36.47) | 0.94 | -7.08 | (-21.72, 7.56) | 0.34 |
| Slope during lockdown | 44.95 | (26.34, 63.56) | <0.001 | 75.34 | (40.34, 110.34) | <0.001 | 24.09 | (9.49, 38.70) | 0.0012 |
| Change in level pre-COVID to post-lockdown | 6.98 | (-18.81, 32.78) | 0.60 | -255.91 | (-304.42, -207.40) | <0.001 | 46.47 | (26.23, 66.72) | <0.001 |
| Change in slope pre-COVID to post-lockdown | -4.86 | (-13.28, 3.57) | 0.26 | 72.10 | (56.25, 87.94) | <0.001 | -17.85 | (-24.46, -11.23) | <0.001 |
| Slope post-lockdown | 36.63 | (30.29, 42.97) | <0.001 | 146.06 | (134.14, 157.98) | <0.001 | 13.33 | (8.35, 18.30) | <0.001 |
| ***JPUH*** |  |  |  |  |  |  |  |  |  |
| Slope pre-COVID | 6.83 | (1.77, 11.89) | 0.01 | 11.91 | (1.24, 22.57) | 0.03 | 8.95 | (4.59, 13.31) | <0.001 |
| Change in level pre-COVID to lockdown | -20.58 | (-56.92, 15.76) | 0.27 | -45.81 | (-122.37, 30.75) | 0.24 | -13.36 | (-44.65, 17.92) | 0.40 |
| Change in slope pre-COVID to lockdown | -1.47 | (-16.16, 13.23) | 0.85 | 3.13 | (-27.83, 34.10) | 0.84 | -6.26 | (-18.92, 6.39) | 0.33 |
| Slope during lockdown | 5.36 | (-9.29, 20.02) | 0.47 | 15.04 | (-15.85, 45.92) | 0.34 | 2.68 | (-9.93, 15.30) | 0.68 |
| Change in level pre-COVID to post-lockdown | -157.95 | (-178.27, -137.63) | <0.001 | -390.11 | (-432.92, -347.30) | <0.001 | -101.40 | (-118.89, -83.91) | <0.001 |
| Change in slope pre-COVID to post-lockdown | 54.80 | (48.17, 61.44) | <0.001 | 128.26 | (114.28, 142.24) | <0.001 | 33.72 | (28.01, 39.43) | <0.001 |
| Slope post-lockdown | 61.63 | (56.64, 66.62) | <0.001 | 140.16 | (129.64, 150.68) | <0.001 | 42.67 | (38.37, 46.97) | <0.001 |
| ***QEH*** |  |  |  |  |  |  |  |  |  |
| Slope pre-COVID | 14.00 | (8.32, 19.69) | <0.001 | 21.19 | (9.26, 33.12) | <0.001 | 13.40 | (9.03, 17.78) | <0.001 |
| Change in level pre-COVID to lockdown | -72.02 | (-112.87, -31.17) | <0.001 | -165.92 | (-251.58, -80.27) | <0.001 | -43.61 | (-75.01, -12.22) | 0.01 |
| Change in slope pre-COVID to lockdown | 10.33 | (-6.20, 26.85) | 0.22 | 36.20 | (1.56, 70.84) | 0.04 | -1.14 | (-13.84, 11.56) | 0.86 |
| Slope during lockdown | 24.33 | (7.85, 40.80) | 0.004 | 57.39 | (22.84, 91.94) | 0.0011 | 12.26 | (-0.40, 24.93) | 0.06 |
| Change in level pre-COVID to post-lockdown | -312.58 | (-335.42, -289.73) | <0.001 | -711.37 | (-759.27, -663.48) | <0.001 | -189.10 | (-206.66, -171.55) | <0.001 |
| Change in slope pre-COVID to post-lockdown | 91.50 | (84.04, 98.96) | <0.001 | 214.41 | (198.77, 230.05) | <0.001 | 52.20 | (46.47, 57.93) | <0.001 |
| Slope post-lockdown | 105.50 | (99.88, 111.11) | <0.001 | 235.60 | (223.83, 247.37) | <0.001 | 65.60 | (61.29, 69.92) | <0.001 |
