# Supplementary material for: Changes in urgent and emergency care activity associated with COVID-19 lockdowns in a sub-region in the East of England: Interrupted times series analyses
Source: PLoS One. 2024 Nov 1;19(11):e0311901. doi: 10.1371/journal.pone.0311901 (PMC11530045; doi:10.1371/journal.pone.0311901)
Supplement: S8 Table — Linear regression models adjusted for time trend and lockdown-trend interaction (model 3). NNUH Norfolk and Norwich University Hospital. JPUH James Paget University Hospital. QEH Queen Elizabeth Hospital. CI confidence interval. (DOCX) [file pone.0311901.s008.docx]

**S8 Table. Temporary changes in numbers of visits to each emergency department during first, second and third lockdown.** Linear regression models adjusted for period, time trend and period-trend interaction (model 3). NNUH Norfolk and Norwich University Hospital. JPUH James Paget University Hospital. QEH Queen Elizabeth Hospital. CI confidence interval

| Type of visit and lockdown period | NNUH | | | JPUH | | | QEH | | |
| --- | --- | --- | --- | --- | --- | --- | --- | --- | --- |
|  | Coefficient | 95% CI | p-value | Coefficient | 95% CI | p-value | Coefficient | 95% CI | p-value |
| All visits |  |  |  |  |  |  |  |  |  |
| 1^st^ lockdown | -64.8 | (-72.8, -56.8) | <0.001 | -47.9 | (-53.5, -42.3) | <0.001 | -32.8 | (-36.8, -28.8) | <0.001 |
| 2^nd^ lockdown | -51.1 | (-64.9, -37.3) | <0.001 | -29 | (-38.7, -19.4) | <0.001 | -12.9 | (-19.8, -6.0) | <0.001 |
| 3^rd^ lockdown | -53.5 | (-62.7, -44.4) | <0.001 | -35.6 | (-41.9, -29.2) | <0.001 | -17.1 | (-21.7, -12.6) | <0.001 |
| Ambulance arrivals |  |  |  |  |  |  |  |  |  |
| 1^st^ lockdown | -14.9 | (-17.8, -12.1) | <0.001 | -4.4 | (-6.3, -2.5) | <0.001 | -5.2 | (-6.8, -3.6) | <0.001 |
| 2^nd^ lockdown | -6.2 | (-11.2, -1.3) | 0.013 | -5 | (-8.3, -1.6) | 0.003 | -1.2 | (-4.0, 1.6) | 0.4 |
| 3^rd^ lockdown | -6.9 | (-10.2, -3.6) | <0.001 | -2.4 | (-4.7, -0.2) | 0.03 | -3.1 | (-4.9, -1.2) | 0.00101 |
| Non-ambulance arrivals |  |  |  |  |  |  |  |  |  |
| 1^st^ lockdown | -49.9 | (-57.0, -42.7) | <0.001 | -43.5 | (-48.4, -38.6) | <0.001 | -27.6 | (-31.1, -24.1) | <0.001 |
| 2^nd^ lockdown | -44.9 | (-57.2, -32.5) | <0.001 | -24 | (-32.6, -15.5) | <0.001 | -11.7 | (-17.8, -5.6) | <0.001 |
| 3^rd^ lockdown | -46.6 | (-54.8, -38.5) | <0.001 | -33.1 | (-38.8, -27.5) | <0.001 | -14.1 | (-18.1, -10.0) | <0.001 |
| Referred by primary care teams |  |  |  |  |  |  |  |  |  |
| 1^st^ lockdown | -5.5 | (-8.3, -2.8) | <0.001 | -0.6 | (-1.6, 0.3) | 0.17 | -0.8 | (-2.3, 0.7) | 0.3 |
| 2^nd^ lockdown | -9.8 | (-14.6, -5.0) | <0.001 | 0.2 | (-1.3, 1.7) | 0.8 | -2.1 | (-4.7, 0.4) | 0.1 |
| 3^rd^ lockdown | -9.2 | (-12.4, -6.0) | <0.001 | -0.5 | (-1.6, 0.5) | 0.34 | -2.7 | (-4.4, -1.0) | 0.002 |
