# Supplementary figures and images for: Changes in urgent and emergency care activity associated with COVID-19 lockdowns in a sub-region in the East of England: Interrupted times series analyses
Source: PLoS One. 2024 Nov 1;19(11):e0311901. doi: 10.1371/journal.pone.0311901 (PMC11530045; doi:10.1371/journal.pone.0311901)

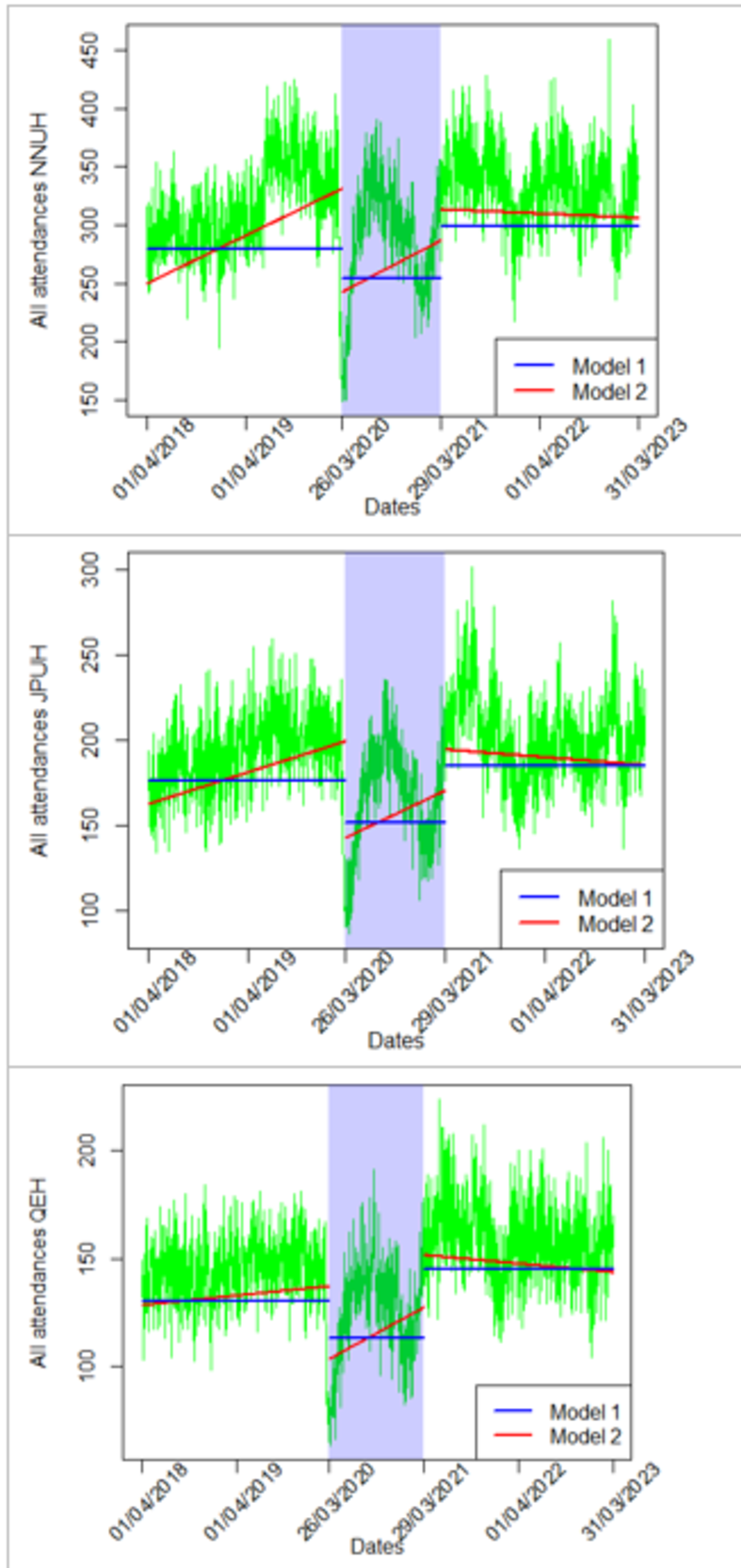

Supplement: S1 Fig — Green lines connect daily observations, blue lines represent Model 1 means, and red lines represent Model 2 levels and slopes. The shaded area represents the lockdown period, the area to the left represents the pre-COVID period and the area to the right represents the post-lockdown period. NNUH Norfolk and Norwich University Hospital. JPUH James Paget University Hospital. QEH Queen Elizabeth Hospital. CI confidence interval. (TIF) [file pone.0311901.s009.tif]

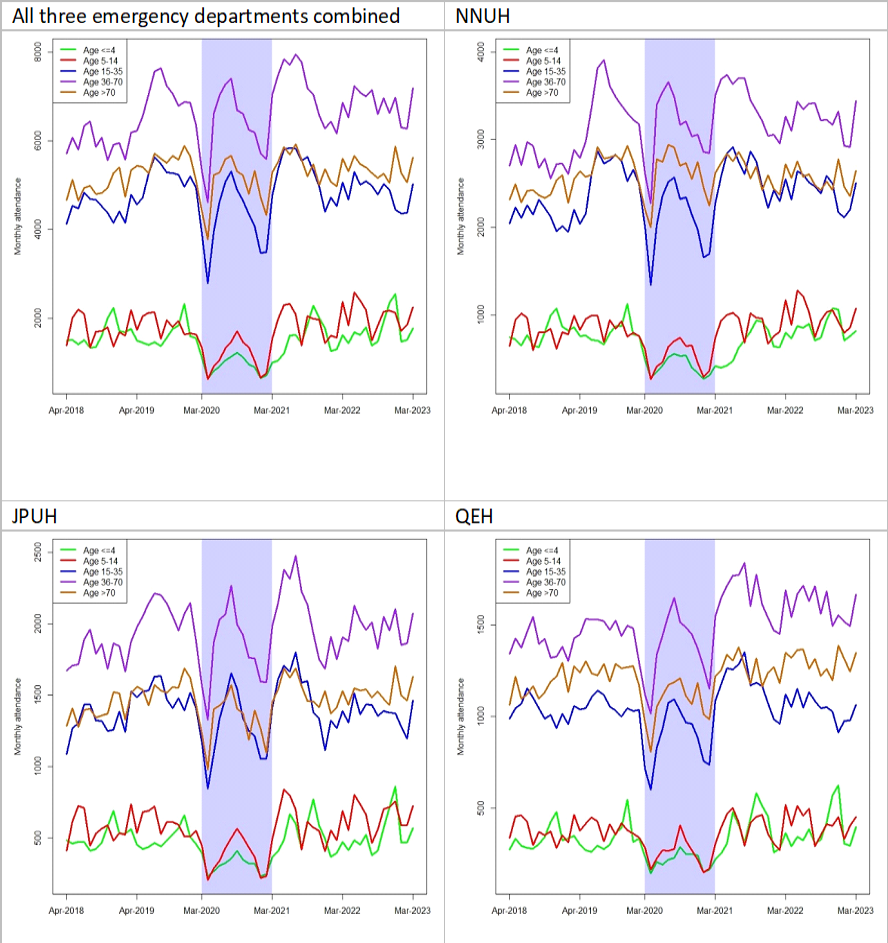

Supplement: S2 Fig — The shaded area represents the lockdown period, the area to the left represents the pre-COVID period and the area to the right represents the post-lockdown period. NNUH Norfolk and Norwich University Hospital. JPUH James Paget University Hospital. QEH Queen Elizabeth Hospital. CI confidence interval. (TIF) [file pone.0311901.s010.tif]
