# Supplementary material for: Changes in urgent and emergency care activity associated with COVID-19 lockdowns in a sub-region in the East of England: Interrupted times series analyses
Source: PLoS One. 2024 Nov 1;19(11):e0311901. doi: 10.1371/journal.pone.0311901 (PMC11530045; doi:10.1371/journal.pone.0311901)
Supplement: S1 File — (DOCX) [file pone.0311901.s011.docx]

**S1: Statistical models**

We applied interrupted time series linear regression models to estimate changes in indicators of urgent and emergency care (UEC) activity during pre-COVID, lockdown and post-lockdown periods. In all models we adjusted for day of the week and month of the year to account for seasonal and within-week variation in activity. Model 1 is the simplest one that compares the average of pre-COVID, lockdown and post-lockdown attendances/calls/average waiting time per day. In Model 2, we estimated changes in level and changes in slope (continuous time trend) in pre-COVID period, and during lockdown and post-lockdown periods. We included interactions between the time trend and the indicator of pre-COVID, lockdown and post-lockdown periods, to estimated how levels and slopes changed during lockdown and post-lockdown periods. In Model 3, we also considered indicators of each of the three lockdown periods, to estimate the temporary effect of each lockdown on UEC activity.

We are discussing the model only for daily attendances. The response variable of interest is the number of daily attendances and hence we calculated the number of daily attendances in each hospital/combined and analysed the day level aggregated data for each hospital separately/combined data. We carried out analyses for all attendances and subgroups based on arrival model. To estimate absolute change in mean daily attendances during lockdown and post-lockdown compared with pre-lockdown period, we used the following linear regression model:

| Model 1: | $Y_{t}=\beta_{0}+\beta_{1}P_{1}+\beta_{2}P_{2}+\boldsymbol{\beta}_{6-11}^{\top}\boldsymbol{W}+\boldsymbol{\beta}_{12-22}^{\top}\boldsymbol{M}+\epsilon$ |
| --- | --- |

where $Y_{t}$ is the daily hospital attendances, $\beta_{0}$ is the mean daily attendances during pre-COVID in reference category day (Friday) and month (April), $\beta_{1}$ is the change in mean daily attendances in the lockdown period compared to pre-COVID period, $P_{1}$ is a binary variables representing lockdown versus pre-COVID, $\beta_{2}$ is the change in mean daily attendances in the post-lockdown period compared to pre-COVID period, $P_{2}$ is a binary variables representing post-lockdown versus pre-COVID periods, $\boldsymbol{W}{=(W_{1},\ldots,W_{6})}^{\top}$ is the set of dummy variables representing days of the week and $\beta_{6},\ldots,\beta_{11}$ are their corresponding regression coefficients, $\boldsymbol{M}{=(M_{1},\ldots,M_{11})}^{\top}$ is the vector of indicator variables that represents the months of the year and $\beta_{12},\ldots,\beta_{22}$ are the corresponding regression coefficients and $\epsilon$ is the random error term.

To estimate the continuous time trend during pre-COVID, lockdown and post-lockdown periods as well as the step change at the start of lockdown and post-lockdown periods compared to the start of study period, we used the following linear regression model:

| Model 2: | $Y_{t}=\beta_{0}+\beta_{1}P_{1}+\beta_{2}P_{2}+\beta_{3}T+\beta_{4}P_{1}T+\beta_{5}P_{2}T+\boldsymbol{\beta}_{6-11}^{\top}\boldsymbol{W}+\boldsymbol{\beta}_{12-22}^{\top}\boldsymbol{M}+\epsilon.$ |
| --- | --- |

Where $Y_{t}$, $P_{1}$, $P_{2}$, $\boldsymbol{W}$, $\boldsymbol{M}$, $\beta_{1}$, $\beta_{2}$, $\boldsymbol{\beta}_{5-10}$ and $\boldsymbol{\beta}_{11-21}$ are same as Model 1, $\beta_{3}$ is the average change per year in daily hospital attendances during pre-COVID period, $T$ represents the number of years from the start study period , $P_{1}T$ and $P_{2}T$ are the interaction between time indicators of lockdown versus pre-COVID and post-lockdown compared to pre-COVID, respectively, $\beta_{4}$ and $\beta_{5}$ are respectively the average change per year in daily attendances in addition to $\beta_{3}$ in lockdown and post-lockdown periods. That is, $\beta_{3}+\beta_{4}$ is the average change per year in daily attendances during lockdown period. Similarly, $\beta_{3}+\beta_{5}$ is the average change per year in daily attendances during post-lockdown period.

Model 3 is that same as Model 2, but with the addition of three binary covariates to represent temporary additional changes in level, during each of the three short-term lockdowns, from the overall lockdown period level.
