# Supplementary material for: Changes in urgent and emergency care activity associated with COVID-19 lockdowns in a sub-region in the East of England: Interrupted times series analyses
Source: PLoS One. 2024 Nov 1;19(11):e0311901. doi: 10.1371/journal.pone.0311901 (PMC11530045; doi:10.1371/journal.pone.0311901)
Supplement: Attachment — Submitted filename: Plos_one_review_change.pdf [file pone.0311901.s012.pdf]

The paper studies the changes in urgent and emergency care activity associated with the COVID-19 lockdowns using time series analysis. The introduction and method section are clear written. The supplementary files provided sufficient and clear details for the time series analysis. My comments are as follows:

1. The authors used data from three hospitals and performed the times-series separately. I suggest authors consider performing analysis after aggregating data together, since an aggregate analysis can identify overarching trends that are consistent across all hospitals
2. Following the last comment, the setting in the article appears to be a classical example in a textbook for mixed-effect models: a variety of collected outcomes were nested in hospitals. Since this approach could provide insights into both individual hospital effects and the overall effect across the system, I wonder if authors consider performing a mixed-effect model analysis for these data.
3. Though authors analyzed the data using three different models, when reporting results, majority of results reported are from the Model 1, and the connection and differentiation between those models are not discussed. I recommend authors discuss the results contrasting these three models.
4. By modeling the hospital data before and after the lock-down, would it be sufficient to conclude that such change is caused by, or associated with, the COVID lockdown? I wonder if authors would consider other confounders or factors that may impact the change in hospital data?

5. In addition to reporting the coefficient values in the text, I recommend authors use tables or plots to report the estimated values for a clearer understanding, especially for readers who may grasp visual information better than textual data.
