# Supplementary material for: Changes in urgent and emergency care activity associated with COVID-19 lockdowns in a sub-region in the East of England: Interrupted times series analyses
Source: PLoS One. 2024 Nov 1;19(11):e0311901. doi: 10.1371/journal.pone.0311901 (PMC11530045; doi:10.1371/journal.pone.0311901)
Supplement: Attachment — Submitted filename: plosone_review.pdf [file pone.0311901.s013.pdf]

The authors use interrupted time series analysis to compare use of urgent and emergency care (UEC) before the onset of the covid-19 pandemic with the use of UEC after the subsequent lockdowns in the U.K. Existing work had shown a reduction in use of hospitals during the early months of the pandemic, but had not yet considered the time after the peak of the covid-19 pandemic and the end of the U.K. lockdowns. They consider a large data set from a consortium of groups providing healthcare across East England and compare the daily attendances at hospitals, ambulances called out, and referrals made to emergency departments before the covid-19 pandemic (2018-2020) and after the subsequent lockdowns (2021-2023). The authors are addressing an interesting problem, but there could be more rigor in the methodology choices and justifications. I have some comments and suggestions for improvement below.

- It would be helpful to include the justification for exclusion of out of area patients and list the number of out of area patients excluded from each region.
- Similarly, it would be helpful to include a justification for analyzing the hospitals separately rather than using some sort of random effect analysis to borrow information across hospitals.
- Provide anova results for table 1 (in supplement if necessary). Provide demographic and descriptive information (gender, ethnicity, number of repeat visits, etc) separately by hospital if the analysis is also being provided separately by hospital.
- In models 1 and 2, why not also model the intermediate period between the lockdowns as a separate portion of the model, making it a three-level variable of pre, during, and post?
- Since the individual level data is available, there seems to be a missed opportunity to consider this question using individual level data, allowing for adjustment for demographic variables, repeated visits, etc. or comparison between groups. If not in the scope of this paper, some discussion of this possibility would be useful.
- In the discussion, reference number 20 does not appear to provide information about the number of doctors and nurses since 2018. Please correct or clarify the reference.
