# Supplementary material for: Changes in urgent and emergency care activity associated with COVID-19 lockdowns in a sub-region in the East of England: Interrupted times series analyses
Source: PLoS One. 2024 Nov 1;19(11):e0311901. doi: 10.1371/journal.pone.0311901 (PMC11530045; doi:10.1371/journal.pone.0311901)
Supplement: Attachment — Submitted filename: Response to editor.docx [file pone.0311901.s015.docx]

Niklas Bobrovitz

Academic Editor

PLOS ONE

Dear Sir

Thank you for your invitation to revise and resubmit this article. We have responded as follows to each of the Editor’s comments. The reviewer’s comments were all favourable and did not require any additional changes. (One reviewer noted that we had not submitted the study data; this was because, as stated in the Data Availability Statement, these data are confidential medical records which the authors are not authorised to share.)

Journal Requirements:

Please review your reference list to ensure that it is complete and correct. If you have cited papers that have been retracted, please include the rationale for doing so in the manuscript text, or remove these references and replace them with relevant current references. Any changes to the reference list should be mentioned in the rebuttal letter that accompanies your revised manuscript. If you need to cite a retracted article, indicate the article’s retracted status in the References list and also include a citation and full reference for the retraction notice.

**Response**. We have added reference 23, and edited the relevant sentence in Discussion. In the previous submission we had written that the article was under review, but it has been published since then.

Additional Editor Comments:

Please address the following comments:

Abstract: Add 95% confidence intervals to the estimates

**Response**: These have been added.

Tables/Figures: Add footnotes describing the abbreviations used in the data displays (e.g., hospital name)

**Response**: We have added ‘CI confidence interval’ and hospital names to the footnotes of all relevant figures and tables in the manuscript and supplementary appendix.

Page 22, line 32: Typo - eliminate word “around”

**Response**: ‘around’ deleted

Page 22, line 37: Type – increasing or decreasing?

**Response**: ‘increasing’ deleted

Yours sincerely

Max Bachmann on behalf of all authors
